# Supplementary material for: Manganese Porphyrin Reduces Oxidative Stress in Vulnerable Parkin-Null Drosophila Dopaminergic Neurons
Source: Antioxidants (Basel). 2025 Aug 22;14(9):1031. doi: 10.3390/antiox14091031 (PMC12466752; doi:10.3390/antiox14091031)
Supplement: Supplementary file 1 [file antioxidants-14-01031-s001.zip › antioxidants-3720963-supplementary.pdf]

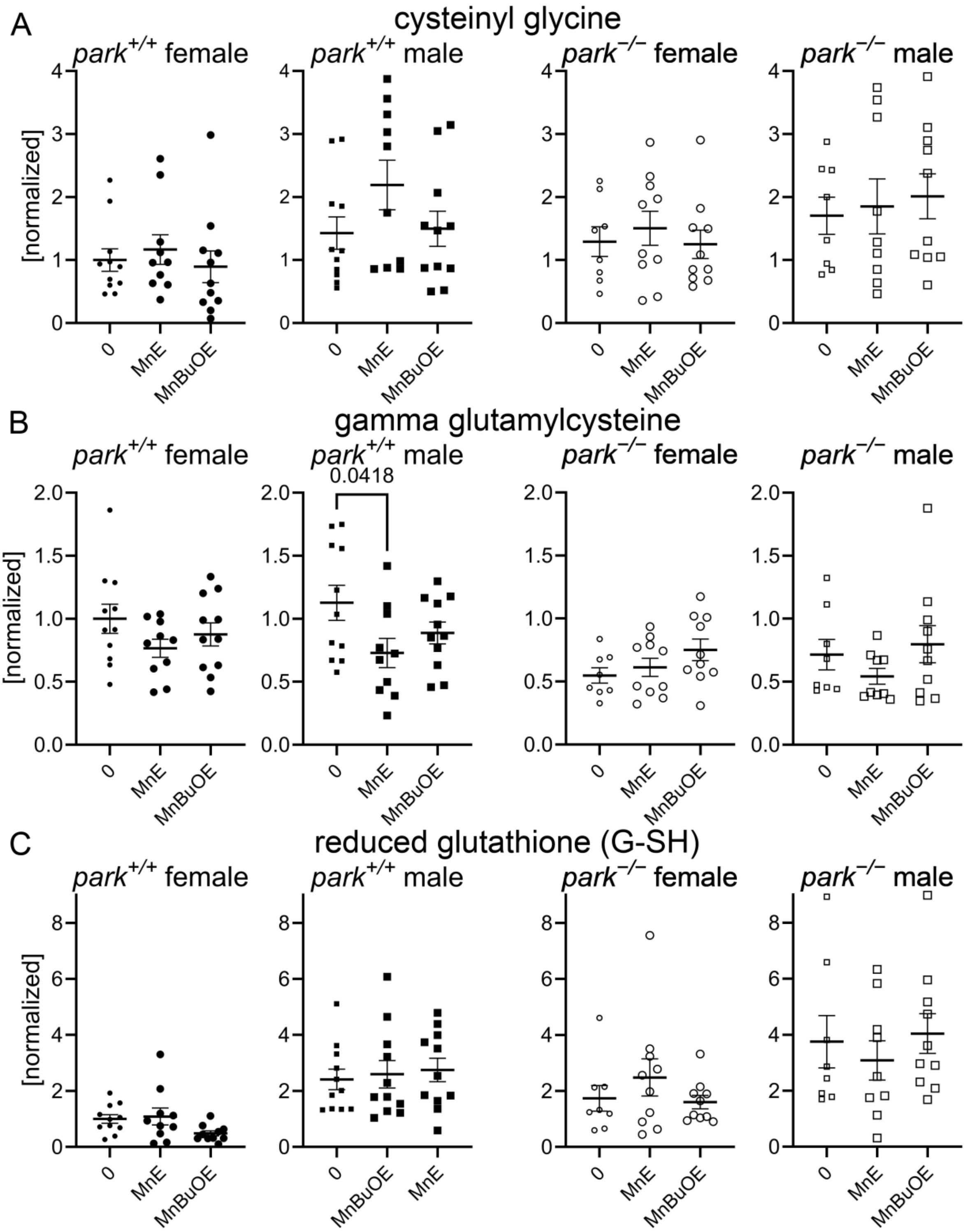

Supplementary Figure S1. Brain levels of cysteinyl glycine, gamma glutamylcysteine, and G-SH are unaffected by MnP administration. Parental stocks were placed on standard food supplemented with 0 or 10 μM MnE or MnBuOE, and newly eclosed control (*park*$^{+/+}$) or parkin-null (*park*$^{-/-}$) progeny were collected and placed on fresh supplemented food. Brain homogenates were harvested on days four to six post-eclosion and cysteinyl glycine (A), gamma glutamylcysteine, and (C) reduced glutathione (G-SH) levels were measured using LC-MS/MS protocols. Data points represent approximately twenty brains pooled into one brain homogenate (n ≥ 8). Circles represent females, and squares represent males. Filled/open circles and squares indicate control/parkin-null flies, respectively. Enlarged circles and squares indicate MnP exposure. One-way ANOVA followed by Dunnett's

multiple comparisons tests were performed to determine the effect of MnP administration. Means, SEM, and Dunnett’s tests p values are indicated.

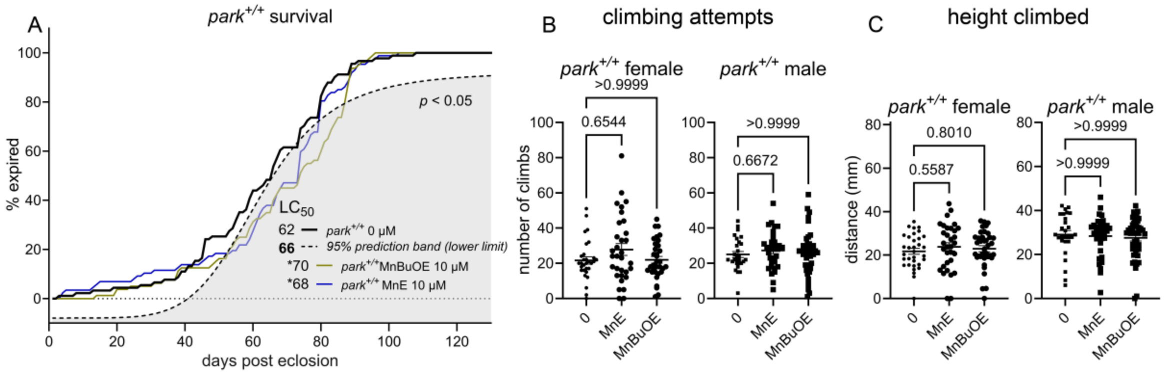

Supplementary Figure S2. MnE and MnBuOE improve survival time for control flies. Parental stocks were placed on food supplemented with 0 or 10 μM MnE or MnBuOE, and newly eclosed control (*park*$^{+/+}$) progeny were placed on fresh, supplemented food. (A) Continuous MnE or MnBuOE exposure improves control fly lifespan. The effects of MnPs are considered to be significant if the corresponding curve’s EC_{50} value is lower than that of the upper prediction limit for untreated flies ($n \geq 62$; $p < 0.05$) [52]. (B,C) Flies were transferred to the Multibeam Activity Monitor on days four to six post-eclosion, when each fly’s position in a vertical tube was recorded every second for twenty minutes. (B) A climbing attempt represents innate motivation to move toward a light source, and average height climbed (C) represents a fly’s ability to move upward in the tube. Data points represent activity of one fly ($n \geq 27$). Circles represent females, and squares represent males. Enlarged circles and squares indicate MnP exposure. One-way ANOVA was performed to determine the effect of MnP administration (B,C). Means, SEM, and p values are indicated.
